# New Insight into the Transcarbamylase Family: The Structure of Putrescine Transcarbamylase, a Key Catalyst for Fermentative Utilization of Agmatine

**DOI:** 10.1371/journal.pone.0031528

**Published:** 2012-02-20

**Authors:** Luis Mariano Polo, Fernando Gil-Ortiz, Angel Cantín, Vicente Rubio

**Affiliations:** 1 Instituto de Biomedicina de Valencia and Centro de Investigación Biomédica en Red de Enfermedades Raras, Valencia, Spain; 2 Instituto de Tecnología Química, Consejo Superior de Investigaciones Científicas-Universidad Politécnica de Valencia, Valencia, Spain; Hungarian Academy of Sciences, Hungary

## Abstract

Transcarbamylases reversibly transfer a carbamyl group from carbamylphosphate (CP) to an amine. Although aspartate transcarbamylase and ornithine transcarbamylase (OTC) are well characterized, little was known about putrescine transcarbamylase (PTC), the enzyme that generates CP for ATP production in the fermentative catabolism of agmatine. We demonstrate that PTC (from *Enterococcus faecalis*), in addition to using putrescine, can utilize L-ornithine as a poor substrate. Crystal structures at 2.5 Å and 2.0 Å resolutions of PTC bound to its respective bisubstrate analog inhibitors for putrescine and ornithine use, N-(phosphonoacetyl)-putrescine and δ-N-(phosphonoacetyl)-L-ornithine, shed light on PTC preference for putrescine. Except for a highly prominent C-terminal helix that projects away and embraces an adjacent subunit, PTC closely resembles OTCs, suggesting recent divergence of the two enzymes. Since differences between the respective 230 and SMG loops of PTC and OTC appeared to account for the differential preference of these enzymes for putrescine and ornithine, we engineered the 230-loop of PTC to make it to resemble the SMG loop of OTCs, increasing the activity with ornithine and greatly decreasing the activity with putrescine. We also examined the role of the C-terminal helix that appears a constant and exclusive PTC trait. The enzyme lacking this helix remained active but the PTC trimer stability appeared decreased, since some of the enzyme eluted as monomers from a gel filtration column. In addition, truncated PTC tended to aggregate to hexamers, as shown both chromatographically and by X-ray crystallography. Therefore, the extra C-terminal helix plays a dual role: it stabilizes the PTC trimer and, by shielding helix 1 of an adjacent subunit, it prevents the supratrimeric oligomerizations of obscure significance observed with some OTCs. Guided by the structural data we identify signature traits that permit easy and unambiguous annotation of PTC sequences.

## Introduction

**Figure 1:**
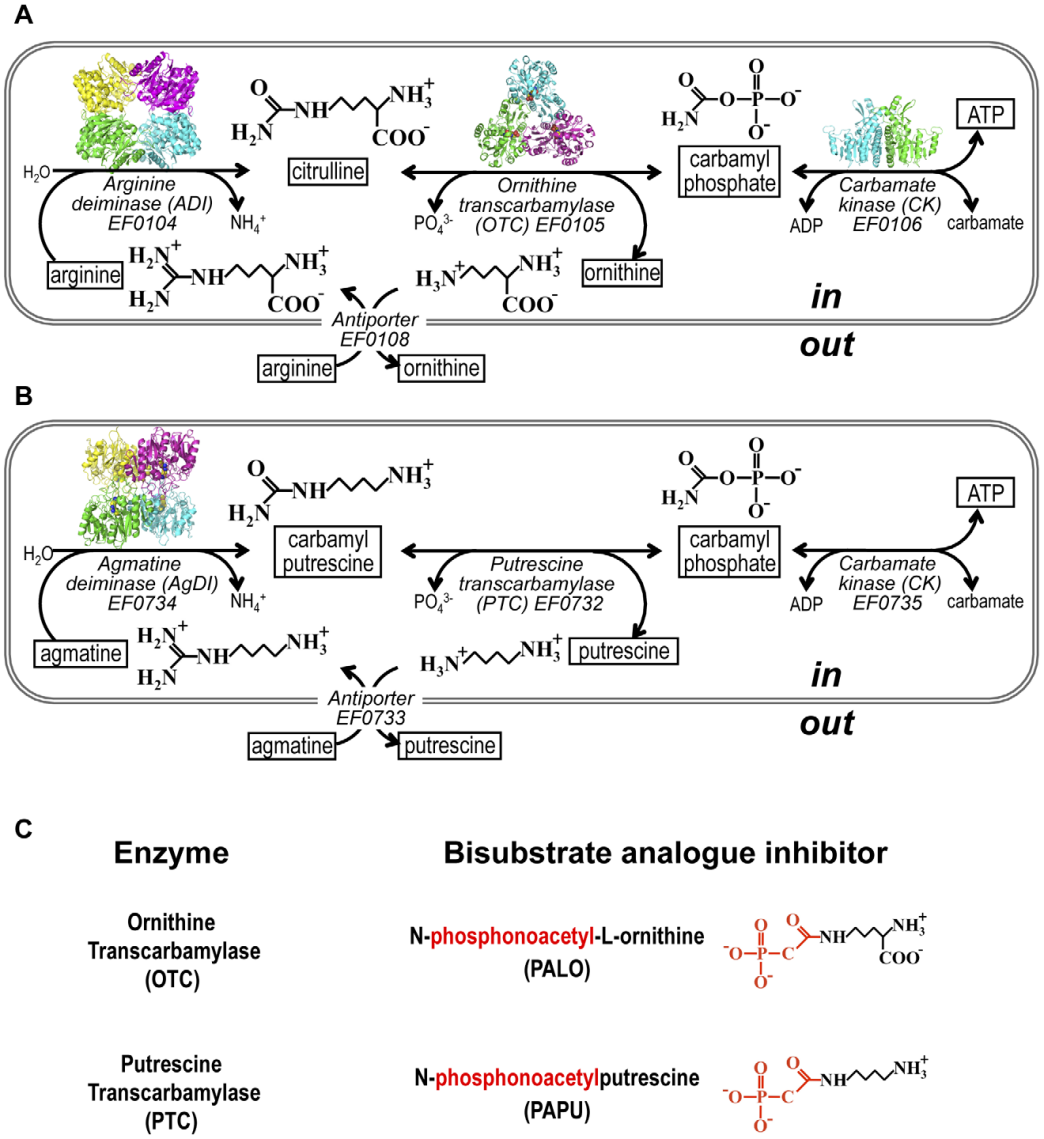
Arginine deiminase and agmatine deiminase pathways and bisubstrate analog inhibitors of OTC and PTC. Representation of agmatine (A) and arginine (B) fermentation routes with the chemical structure of the compounds involved and the cartoon diagrams of the known protein structures: *E. faecalis* AgDI (2JER), *P. aeruginosa* ADI (1RXX), *E. coli* OTC (2OTC) and *E. faecalis* CK (2WE5). (C) Inert bisubstrate analog inhibitors for OTC and for PTC, PALO and PAPU, respectively. The carbamylphosphate moiety of each inhibitor is colored red and the putrescine or the ornithine moiety in black.

Transcarbamylation is a key enzymatic function for essentially all life forms. Carbamylphosphate (CP), an energy-rich compound that may have originated in the prebiotic world [1], is the source of a carbamyl group for incorporation into organic molecules that are crucial for virtually all life forms, like pyrimidines or arginine [2]. Transcarbamylases are the enzymes that carry out this work, by carbamylating an amine using as donor the carbamyl group of CP. These carbamyl group incorporations are mediated by aspartate transcarbamylase (ATC) or ornithine transcarbamylase (OTC) and its variant forms using αN-acetyl- or αN-succinyl-L-ornithine [3], [4]. However, transcarbamylases are not only used as biosynthetic enzymes. Catabolic transcarbamylases exploit the reversibility of the transcarbamylation reaction, deriving energy fermentatively from carbamyl-group containing organic compounds [5]–[12]. Thus, many microorganisms catabolize arginine by a pathway (the arginine deiminase pathway) (Figure 1A) using a catabolic OTC [5], [6]. Similarly, agmatine, the decarboxylated analogue of arginine, can be used as a fermentative source of ATP utilizing putrescine transcarbamylase (PTC) in this process (Figure 1B) [7]–[9]. Such process may endow with resistance to acidic pHs to the organisms hosting it, like the caries-producing *Streptococcus mutans* [13] or the food pathogen *Listeria monocytogenes* [14]. In another similar fermentative process, allantoin, a purine catabolite of many mammals, or its degradation product oxalurate, can be utilized as energy source in a pathway involving oxamate transcarbamylase [10]–[12]. In all these cases the transcarbamylase makes CP from the carbamyl group-containing compound and phosphate, and this CP is then used for phosphorylating ADP in a reaction catalyzed by the enzyme carbamate kinase (Figure 1).

Surprisingly, despite the fact that transcarbamylases catalyzing CP production from carbamyl group-containing compounds have been known for many years already, only one catabolic transcarbamylase, OTC, was studied in detail (see for example [15], [16]). Quite limited information, which does not include structural data, exists, for example, about PTC [7], [9], despite the fact that inhibition of agmatine catabolism in *S. mutans* might be of value in caries prevention [17]. Similarly, virtually nothing is known about oxamate transcarbamylase other than its occurrence in a number of microorganisms like *Enterococcus faecalis* [10], [11], a coccus that can cause opportunistic infections and is sensitive to very few antibiotics [18], and which also has the agmatine deiminase and arginine deiminase catabolic pathways [5], [8], [11]. In the case of oxamate transcarbamylase not even its gene has been identified, whereas the gene for PTC was identified [19], [20] and positively proven to encode this enzyme just few years ago [9].

We have undertaken the task of characterizing structurally catabolic transcarbamylases that are presently not well understood. We report here the crystal structure at 2.5 Å resolution of PTC from *E. faecalis* bound to a bisubstrate analogue inhibitor [9], N-(phosphonoacetyl)-putrescine (PAPU, Figure 1C). This transcarbamylase has the additional interest of not being completely specific [7] (as conclusively proven here), being able to use L-ornithine as a poor substrate in addition to using its genuine substrate putrescine, raising the issue of whether OTC and PTC evolved from a common ancestor not differentiating between L-ornithine and its decarboxylated analogue putrescine or whether PTC derives from OTC in an as yet incomplete process of changing specificity from ornithine to putrescine. The structural closeness of PTC to the OTC of *Pyrococcus furiosus* would appear to support the second possibility. In any case, we clarify here which structural elements determine the preference of the enzyme for putrescine. Furthermore, our present determination of the crystal structure at 2.0 Å resolution of the complex of PTC with the corresponding bisubstrate analogue for ornithine use, δ-N-(phosphonoacetyl)-L-ornithine (PALO, Figure 1C), explains why this enzyme can use ornithine. These findings shed light on how PTC became able to use an amine not having a carboxylate group, providing hints on how to engineer transcarbamylases to change their specificity. Such engineering is illustrated here by our reversion of the substrate preference of PTC, rendering the enzyme a better OTC and a much poorer PTC.

On the basis of the structure we highlight here some sequence traits that appear diagnostic of PTCs and that may ease recognition of this enzyme in sequence databases. Among these traits, one concerns an unexpected structural feature, the presence of a very prominent C-terminal helix that interlinks adjacent subunits in the PTC trimer. Sequence comparisons indicate that this helix, which has not been found in any other transcarbamylase, is constant among PTCs. We prove here by in silico studies and by helix deletion and experimental investigations (including X-ray crystallography of the truncated enzyme) that this C-terminal helix plays paramount roles in trimer stabilization and in the prevention of formation of supratrimeric oligomers similar to those seen with some OTCs [15], [16], [21]. This raises the question of which is the significance of higher oligomer formation among transcarbamylases.

Another intriguing feature requiring functional clarification is our present finding of one Ni atom binding at the trimer threefold axis, at a site similar to the ones found in the catabolic OTCs from *Lactobacillus hilgardii* [16] and *Giardia lamblia* [22]. The finding of metals binding in these OTCs and the report that rat OTC is a conspicuous cadmium-binding liver protein [23] warrants the study of the significance of the metal interactions of transcarbamylases.

## Results and Discussion

### PTC genuinely uses ornithine as substrate

**Figure 2:**
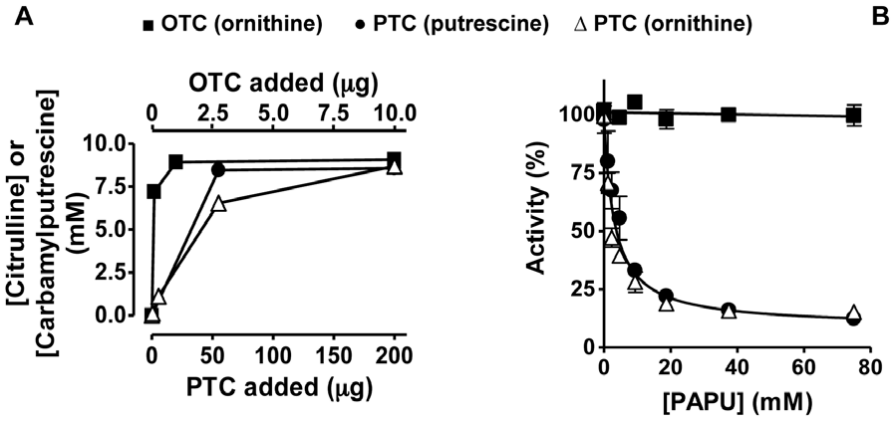
PTC carbamylates ornithine in addition to putrescine. (A) Approach to equilibrium in the carbamylation of ornithine (open symbols) using either OTC (squares) or PTC (triangles) from *E. faecalis* as catalyst and comparison with the equilibrium for putrescine carbamylation catalyzed by PTC (closed symbols). Tubes containing the indicated amounts of either OTC or PTC in 0.25 ml of 0.1 M Tris-HCl pH 8.5, 0.4 mg/ml bovine serum albumin, 10 mM carbamylphosphate, and 10 mM of either ornithine or putrescine (as indicated), were incubated 10 min at 37°C. Then 0.1 ml of cold 20% (w/v) trichloroacetic acid was added, and the amount of citrulline or carbamylputrescine, respectively, was determined [9]. The results show the amount of these ureido compounds in the 0.25-ml incubation mixtures. (B) Inhibition by increasing concentrations of PAPU of the transcarbamylase activities of *E. faecalis* PTC using putrescine (closed circles) or ornithine (open triangles) as substrates, and lack of inhibition of *E. faecalis* OTC (open squares). Activities are given as a percentage of the activities in the absence of PAPU. A single curve has been fitted to the results observed for PTC activity with both putrescine and ornithine as substrates.

We conclusively confirm a previous report [7] that PTC uses L-ornithine as a poor substrate. The possibility that this activity were due to traces of putrescine contaminating the ornithine was excluded by bringing the reaction with ornithine to equilibrium with large amounts of recombinant PTC, comparing the results with those obtained with pure recombinant OTC (from *Enterococcus faecalis*) (Figure 2A). The same equilibrium point was attained with both enzymes, judged by measuring citrulline, corresponding to ∼90% conversion of ornithine to citrulline. If the activity had been due to traces of contaminating putrescine a much lower concentration of the carbamylated product (carbamylputrescine and citrulline give similar color in the Archibald reaction used [9]) would have been obtained at equilibrium with PTC. The alternative artifactual possibility for ornithine use by PTC, that the PTC preparation were contaminated by traces of OTC, was excluded (Figure 2B) by showing that the PTC-specific bisubstrate analogue inhibitor PAPU inhibited similarly the activities of PTC with putrescine and with ornithine whereas this compound did not substantially inhibit citrulline synthesis by purified OTC. A further definitive proof for the responsibility of PTC for using ornithine was provided by mutation of one residue of the active center of this enzyme. The R54G mutant prepared by site-directed mutagenesis (see Materials and Methods) did not exhibit any PTC or OTC activity despite the fact that it was highly soluble and was purified to homogeneity similarly to the wild-type enzyme (detection limit of the activity assay, 1,000-fold lower activity than wild-type PTC). Indeed, the determination of the crystal structure of PTC bound to the bisubstrate inhibitor for ornithine utilization, PALO (see below), provided a direct proof that PTC is able to handle ornithine as substrate.

### PTC crystals and diffraction data

Crystal structures (Table 1) of PTC bound either to PAPU (PTC-PAPU) or PALO (PTC-PALO) at 2.5 and 2.0 Å resolution, respectively, were obtained. Phasing of the diffraction data for the PTC-PAPU crystal, using molecular replacement with a polyalanine model of one subunit of *Pyrococcus furiosus* OTC [21] (pfOTC; 43% identity and 74% identity+similarity for the 315-residue sequence overlapping in pfOTC and PTC), yielded two PTC protomers in the asymmetric unit. Molecular replacement with the refined model for one PTC-PAPU protomer yielded two trimers in the asymmetric unit of the PTC-PALO crystal. The same approach was used for phasing of the crystal (diffracting at 1.6 Å resolution) of PTC lacking the C-terminal helix and bound to PALO (see below and Table 1). All models had excellent R_free_ values and exhibited good stereochemistry, although M125 and L270, which are involved in putrescine binding (see below) are outliers in the Ramachandran plot, similarly to the equivalent OTC residues (L163 and L304 of human OTC, hOTC; unless indicated, hOTC will be used in all the comparisons with PTC because of its close structural similarity with it and the report of the structure of the hOTC-PALO complex [24]). The structure also encompasses two *cis*-proline residues, P248 and P271, which are also involved in the putrescine site.

**Table 1:** X-Ray Data and Structure Refinement Statistics.

	PTC-PAPU	PTC-PALO	Truncated PTC-PALO
Data collection			
ESRF Beamline	BM16	ID23-2	ID14-4
Wavelength (Å)	0.980	0.873	0.979
Space group	P6_3_22	P1	P6_3_22
Unit cell a, b, c (Å)	117.2, 117.2, 225.3	81.5, 81.7, 82.3	90.0, 90.0, 184.3
α, β, γ (°)	90, 90, 120	105, 103, 101	90, 90, 120
Resolution range (Å)^a^	30–2.50	30–2.00	40-1.59
	(2.59–2.50)	(2.10–2.00)	(1.67–1.59)
Reflections, total/unique	692,675/32,509	194,037/114,553	611,850/60,518
Completeness (%)^a^	100 (100)	87.5 (79.5)	100 (99.8)
I/σ^a^	35.1 (9.5)	10.9 (1.8)	5.7 (2.1)
*R*_sym_ b (%)^a^	11.5 (42.8)	5.5 (42.5)	8.2 (36.6)
Refinement Statistics			
Resolution range	23–2.50	30–2.00	30-1.59
*R*-factor/*R*_free_ c	18.7/21.6	19.6/23.7	16.2/18.0
Molecules and atoms refined			
Polypeptide chains	2	6	1
Protein atoms	5,422	15,480	2,446
PAPU or PALO	2	6	1
RMSD^d^ bonds (Å)/angles (°)	0.011/1.16	0.008/1.01	0.007/1.13
Average *B*-factor (Å^2^)			
Protein	19.8	33.8	12.7
PAPU or PALO	11.1	30.2	8.5
Ramachandran Plot^e^ (%)			
Favored	93.3	92.2	92.6
Allowed	6.1	7.1	6.4
Generously allowed	0	0	0
Disallowed	0.7	0.7	0.7

a Values in parenthesis are data for the highest resolution shell.

b *R*_sym_ = Σ*I*−<*I*>/Σ*I*, where *I* is the observed intensity and <*I*> the average intensity.

c *R*-factor = Σ*_h_* ∥*F*_obs_|−|*F*_calc_∥/Σ*_h_* |*F*_obs_|, where |*F*_obs_| and |*F*_calc_| are observed and calculated structure factors amplitudes for all reflections (*R*-factor). *R*_free_, R based on 5% of the data, withheld for the cross-validation test.

d RMSD: root mean square deviation.

e Using PROCHECK.

### The PTC subunit

**Figure 3:**
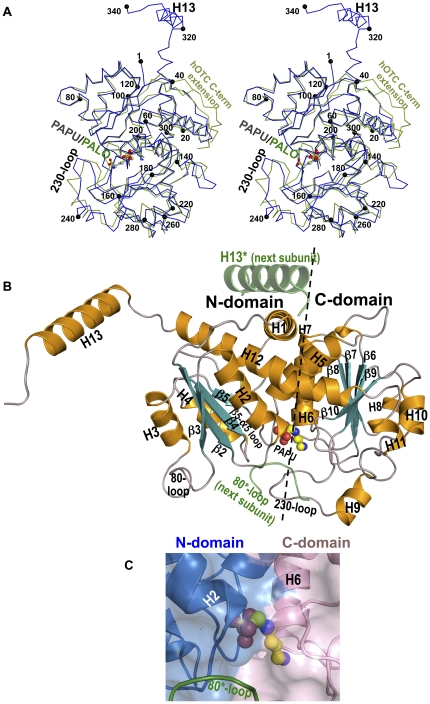
The PTC protomer structure. (A) Superimposition of Cα trace of the subunit of *E. faecalis* PTC (blue) with that of hOTC (green, PDB file 1OTH). Black spheres mark every twentieth residue. PAPU (C atoms colored grey) and PALO (C atoms colored green) are depicted in ball and stick representation, with O and N atoms in red and blue, respectively. (B) Cartoon representation of a PTC protomer of PTC-PAPU, with the ligand in ball and stick representation. Helices and β-strands are colored orange and turquoise, and labeled, and loops are grey and are labeled when highly relevant. Helix 13 and the 80-loop of an adjacent subunit are shown colored green and semitransparent. They are marked with asterisks. (C) Close-up of PAPU bound to PTC. N- and C-domains are shown as transparent surfaces colored blue and pink, respectively, with secondary structure elements shown below in cartoon representation. The 80-loop* from the adjacent subunit is shown in green and marked.

The 8 subunits in the asymmetric units of the PTC-PAPU and PTC-PALO crystal structures are essentially identical (root mean square deviation, rmsd, for superimposition of their Cα atoms, 0.15–0.54 Å). The largest differences are due to changes in the active center associated with the binding of the different bisubstrate analogue inhibitors PAPU or PALO (see below). Each subunit has a characteristic shape consisting of a hemispheric subunit body and a projecting tail (Figures 3A and 3B) formed by a C-terminal helix (helix 13) that, as shown below, is a characteristic and exclusive PTC feature. The subunit body presents the classical transcarbamylase fold [3], consisting of two domains of similar size and gross fold, the N-domain (PTC residues 1–130 and 301–315), and the C-domain (residues 131–296), corresponding respectively to the polar and equatorial domains of ATC [3] and to the CP and ornithine domains of OTC [15]. These domains are folded as open αβα sandwiches nucleated by parallel β sheets (sandwich composition and β-sheet topologies, α_3_β_5_α_2_ and 1-5-4-2-3, respectively, for the N-domain; and α_3_β_5_α_4_ and 8-7-6-9-10 for the C-domain) (Figure 3B). The two domains are interconnected by helices 5 and 12, with additional interdomain gluing being provided by helix 1, which runs transversely over helices 5 and 12, at the interdomain divide on the convex face of the subunit. The flat face of the hemispheric subunit buries the bisubstrate analog inhibitor at the interdomain divide (Figure 3B). Characteristically for transcarbamylases, the β sheets C-edges of both domains look towards this flat face, and the loops emerging from the β strands C-ends participate in the active center (Figures 3B and 3C), including the β5-α5 loop (residues 124–130), the 230-loop (residues 227–253, including helix 9), the β10-α11 loop (residues 269–279), and the 80-loop* (residues 74–85*) of an adjacent subunit (an asterisk denotes an element from an adjacent subunit). The participation of two subunits in the active center supports the observation made with aspartate transcarbamylase that the trimeric architecture (see next section) is essential for activity [25].

**Figure 4:**
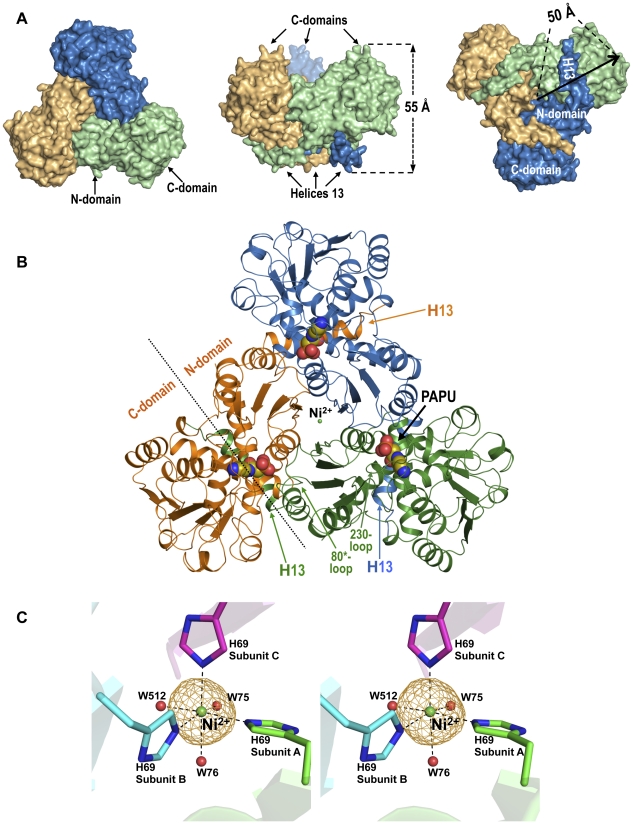
Structure of PTC trimer. (A) Surface representations of the trimer along the threefold axis from the concave or convex faces (left and right, respectively) or with the threefold axis vertical and the concave face up (center). Each protomer is in a different color. (B) Cartoon representation of the PTC trimer viewed along its threefold axis with its concave face close to the viewer. PAPU is shown in space-filling representation. Some elements are labeled, and in one protomer the boundary between the N- and C-domains is signaled with a broken line. (C) Stereoview representation of the electron density omit map for the Ni ion (2.5σ) in one trimer of PTC-PALO, showing the coordinated histidine and water (marked W75, W76 and W512) molecules.

Although the composition, topology, and even the length of the secondary structure elements of the PTC subunit are highly similar to those of the corresponding domains of pfOTC and hOTC (rmsd for superimposition of the subunits of the PTC-PAPU subunit excluding the C-terminal α-helix, with those of hOTC-PALO, 1.27–1.29 Å for 299–300 Cα atoms), there are some differences with these enzymes (illustrated in Figure 3A for hOTC). These differences affect particularly the specificity-determining 230-loop which encompasses helix 9, and the C-terminus of the subunit, where helix 12 is 1.5-turn longer than in OTCs and is connected via a 4-residue linker with the already mentioned extra C-terminal helix (helix 13). The latter 5-turn helix (residues 320–337) is exclusive of PTC, it is highly prominent, and it is oriented approximately at a right angle with respect to helix 12 (Figures 3A and 3B). Helix 13 is projected away from the subunit, landing on an adjacent subunit, with which it makes extensive interactions (Figure 4). Although hOTC also exhibits a C-terminal extension that is not present in bacterial or achaeal OTCs [24] (Figure 3A), this extension only has 7 residues instead of the 25 residues of the PTC extension, and it is merely a loop that does not project away from the subunit, folding over helix 1 of the same subunit. Nevertheless, as discussed below, this extension and the C-terminal helix of PTC may share some functional similarity.

Comparison (using PDBeFold [26], http://www.ebi.ac.uk/msd-srv/ssm/cgi-bin/ssmserver) of the structure of the PTC subunit with all the protein structures in the Protein Databank (http://www.pdb.org/pdb/home/home.do) identified as the closest structures to that of the PTC subunit those of the subunits of pfOTC, *Thermotoga maritima* OTC (tmOTC) and hOTC, (Protein Databank files 1PVV, 1VLV and 1OTH, respectively). This closeness with OTCs supports our previous suggestion [9] that PTC might have evolved from an OTC. These three closest OTCs, as well as PTC, lack an internal helix (called helix 10′ or 9a) that is found in some bacterial OTCs of the α-type [27], such as the *E. coli* [28] and *P. aeruginosa* [15] OTCs, two OTCs that are highly represented in structural databases because of their early structural study.

### The PTC trimer

The application of the crystal symmetry to the two protomers found in the asymmetric unit of the crystal of PTC-PAPU generates two trimers. These trimers are essentially identical to the two trimers found in the asymmetric unit of the crystal of PTC-PALO (rmsd for superimposition of their Cα atoms, 1.05–1.37 Å). Furthermore, except for the PTC-exclusive C-terminal helix, the PTC trimer closely resembles the OTC basic trimer (Figure S1) (rmsd, 1.31–1.37 Å for 894–900 superimposed Cα atoms of PTC-PAPU with hOTC-PALO, PDB file 1OTH). The trimer is roughly shaped (Figure 4A) like a triangular shallow cup of ∼55 Å deepness and ∼50 Å radius from center-to-vertexes. The C-domains (Figures 4A, B) occupy the three vertexes and protrude from the cup concave face, the face that hosts the flat faces of the three subunits and the active centers. The N-domains sit next to the threefold axis and provide all the intersubunit contacts excepting those mediated in PTC by helix 13. Interestingly, a mass of electron density fitting one Ni ion and making the expected coordinative contacts for such ion is found at the threefold axis in the trimer convex face in all the PTC crystals studied here (Figure 4C). The Ni, which is octahedrally coordinated to three H69 N atoms (one per subunit) and three O atoms of fixed water molecules (Figure 4C), may have derived from the Ni-chelate column used for PTC purification. Ni was found binding in the same site of *Lactobacillus hilgardii* catabolic OTC [16]. Given the octahedral coordination that is characteristic for metals of the transition group II of the periodic table, including Cd [29], these observations of a metal site in PTC and in at least one OTC might perhaps explain the reported Cd avidity of liver OTC [23], rendering important to examine the significance of this metal site in these enzymes.

### A C-terminal helix links adjacent subunits in the PTC trimer

**Figure 5:**
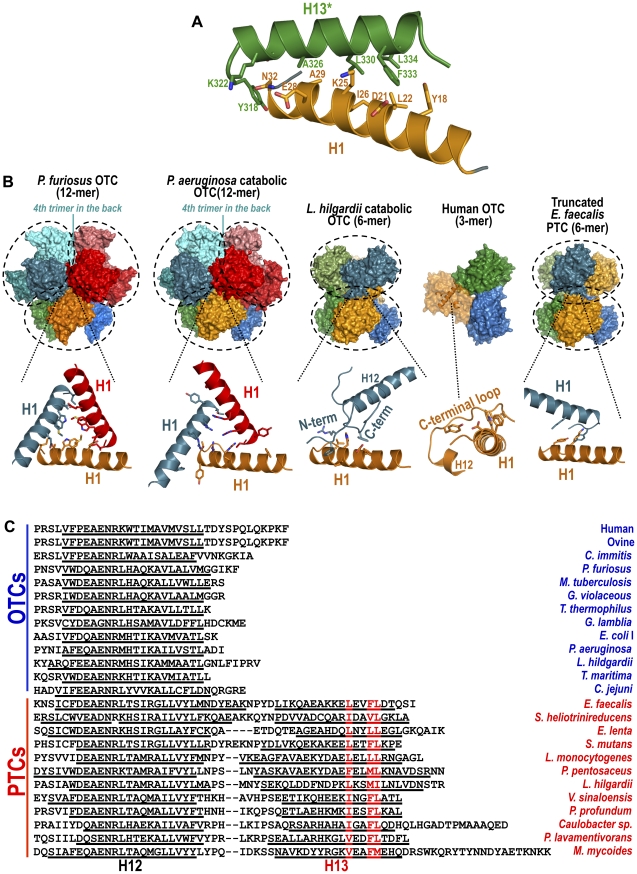
Helix 1, helix 13, and presence or absence of supratrimeric oligomerization in OTC and PTC. (A) Cartoon representation of helix 1 (in orange) and H13* of the adjacent subunit (in dark green) showing residues involved in the contacts between these helices. (B) Oligomeric structures of (from right to left): pfOTC (PDB file 1PVV), *Pseudomonas aeruginosa* OTC (1DXH), *Lactobacillus hilgardii* OTC (2W37), hOTC (2OTH) and *E. faecalis* truncated PTC. For each structure, the biological assembly is shown in the top part, with each basic trimer encircled in a broken line, focusing in the bottom part on the interactions of helix 1. In hOTC the surface of a subunit is shown in semitransparent representation and helix 1 in illustrated in cartoon form. The color code of the helices shown in the bottom part corresponds to that of the subunits involved in the interactions shown. (C) Sequence alignment of the C-terminal portions of OTCs of known 3-D structure and of representative known or putative PTCs (annotated as such in UniProt and NCBI Protein databases). Red lettering marks the PTC sequence signature identified in helix 13. Underlining marks experimentally observed (OTCs and *E. faecalis* PTC) or predicted (other PTCs) α-helices. Secondary structure was predicted with SOPMA (http://npsa-pbil.ibcp.fr/cgi-bin/npsa_automat.pl?page=npsa_sopma.html). PDB files are the following for OTCs: human, 1OTH; *Ovis aries*, 1FB5; *Coccidioides immitis*, 3SDS; *P. furiosus*1PVV; *Mycobacterium tuberculosis*, 2I6U; *Gloeobacter violaceus*, 3GD5; *Thermus thermophilus*, 2EF0; *Giardia lamblia*, 3GRF; *E. coli*, I form, 2OTC; *P. aeruginosa*, 1DXH; *L. hilgardii*, 2W37; *Thermotoga maritima*, 1VLV; *Campylobacter jejuni*, 3TPF. For PTCs, UniProt or NCBI Protein database entries are the following: *E. faecalis*, Q837U7; *Eggerthella lenta*, C8WMM1; *S. mutans*, Q8DW19; *L. monocytogenes*, D2P024; *Pediococcus pentosaceus*, Q03HM9; *L. hilgardii*, C0XJB3; *Vibrio sinaloensis*, E8M734; *Mycoplasma mycoides*, Q6MSR6; *Slackia heliotrinireducens*, YP_003143629.1; *Photobacterium profundum*, YP_133569.1; *Caulobacter sp.*, YP_001684585.1; *Parvibaculum lavamentivorans*, YP_001412420.1.

Helix 13 sits on the next subunit in the anticlockwise direction (looking from the trimer convex face along the threefold axis, Figure 4A, right panel), marking in this subunit the interdomain divide. It covers helix 1 and runs approximately antiparallel to it (Figures 3B and 5A). In this way, helix 13* forms a protruding linear ridge in the convex face of each subunit (Figure 4A, center and right panels), running transversely relative to the radii joining the threefold axis to the C-domains. The discontinuous wall formed by these three ridges encircles the three N-domains of the trimer (Figure 4A right panel and Figure 4B) and gives the PTC trimer a very characteristic appearance. The presence of this helix and the shielding by it of helix 1 (Figures 3B, 4B and 5A) is a key differential feature of PTC relative to any other transcarbamylase (exemplified in Figure 5B). Furthermore, helix 13 appears to be a constant PTC feature. Thus, the sequences annotated as PTCs in protein databases (UniProt, http://www.uniprot.org/; NCBI Protein, http://www.ncbi.nlm.nih.gov/protein) have C-terminal extensions sharing a predicted high propensity for α-helix formation in the region corresponding to helix 13 of *E. faecalis* PTC (underlined in the PTC sequences of Figure 5C). Furthermore, these extensions share a weakly diagnostic but constant signature sequence ^330^(L/f/v/i/m)XX(F/Y/L/M/v)(L/i/m) (capitals indicate predominance, small case, occurrence with lower frequency, X, any residue; residue numbering of *E. faecalis* PTC; the signature is in red in Figure 5C). Therefore, helix 13 is a prominent element that appears constant, characteristic and exclusive of PTCs.

### Effects of C-terminal helix deletion

A major role of the C-terminal helix appears to be the stabilization of the PTC trimer. Thus, ∼750 Å^2^ and ∼610 Å^2^ of helix 13 and of the other subunit body are buried at each helix-body interface (estimated with a probe of radius 1.4 Å). This interface involves a hydrophobic patch including the conserved residues of the helix signature (Figure 5A). Furthermore, the PISA server (http://www.ebi.ac.uk/msd-srv/prot_int/cgi-bin/piserver [30]) gave a much higher energy cost for dissociation to monomers of the PTC trimer (ΔG = 59 kcal/mol) than of the trimer lacking this helix (∼14.6 kcal/mol) or of the trimer of hOTC (26.4 kcal/mol), an enzyme that has no C-terminal helix.

**Figure 6:**
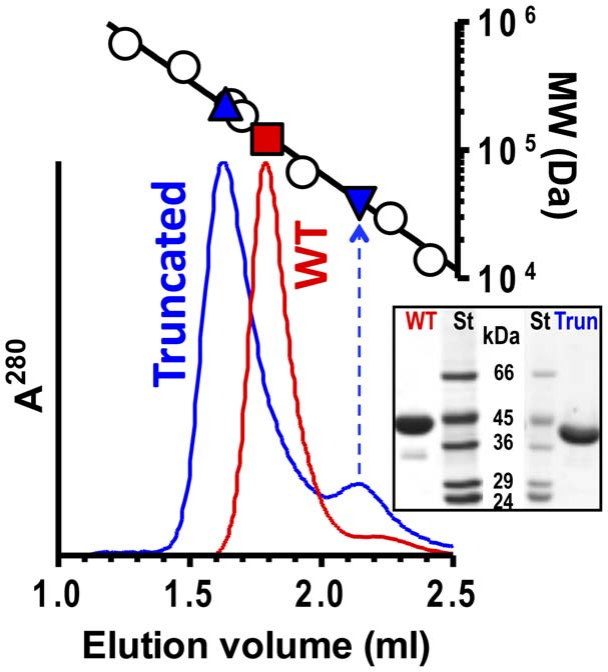
Size exclusion chromatography of wild type and truncated PTC. Lower panel: Optical density at UV^280^ of the effluent of the Superdex 200HR column after injection of wild-type *E. faecalis* PTC (red) or of this enzyme lacking helix 13 (blue). Top, semilogarithmic plot of molecular mass versus elution volume for marker proteins (see Materials and Methods) and for the observed peaks if they corresponded to trimers or wild type PTC (red square, sequence-deduced mass for a trimer, 120.3 kDa), or to truncated PTC hexamers (blue triangle, sequence-deduced mass for the hexamer, 230 kDa) and monomers (blue inverted triangle; sequence-deduced mass, 38.3 kDa). The inset shows Coomassie stained SDS-PAGE of purified wild type (WT) and truncated (Trun) PTC, together with marker proteins (St) with masses in kDa.

We confirmed the decreased stability of the trimer of PTC lacking the C-terminal helix by deleting this helix. The protein with this deletion (generated by introducing a stop codon at position 318) was purified normally from *E. coli* and gave nearly normal enzyme activity (619±42 U/mg, compared with 670±68 U/mg for wild-type PTC). This excludes massive rapid dissociation of PTC trimers, since transcarbamylases must be trimeric to be active [25]. Nevertheless, a significant although small fraction of truncated PTC was eluted as monomers from a size exclusion chromatography column (Figure 6). Since no monomer peak was observed with wild type PTC (Figure 6), this indicates that the truncated enzyme has an increased tendency to dissociate, as expected. The size exclusion chromatography studies also revealed the elution of the majority of the truncated enzyme at a volume expected for hexamers, whereas wild-type PTC was eluted as trimers (Figure 6). Thus, the C-terminal helix prevents the association of PTC into hexamers. Supratrimeric architectures have been reported for pfOTC, which is a dodecameric tetramer of trimers (Figure 5B, leftmost panel) [21], and for the catabolic OTCs of *Pseudomonas aeruginosa* (again a dodecamer) [15] or *L. hilgardii* (a hexamer) [16] (Figure 5B, second and third panels from the left). Helix 1, the helix that is buried in PTC by helix 13, is centrally involved in the intertrimeric contacts that lead to the hexamer or dodecamers in these supratrimeric OTCs [15], [16], [21] (Figure 5B, details). We confirmed the involvement of helix 1 in the formation of the hexamers of PTC lacking helix 13 by crystallizing this truncated enzyme. The crystal structure at 1.6 Å resolution confirmed hexamer formation mediated by intertrimeric contacts involving helix 1 (Figure 5B, rightmost panel and detail below it, and S2). Since these contacts are abolished in wild-type PTC by the presence of helix 13, a function of this helix appears to be to prevent the formation of supratrimeric architectures.

### The active center and the discrimination between putrescine and ornithine

**Figure 7:**
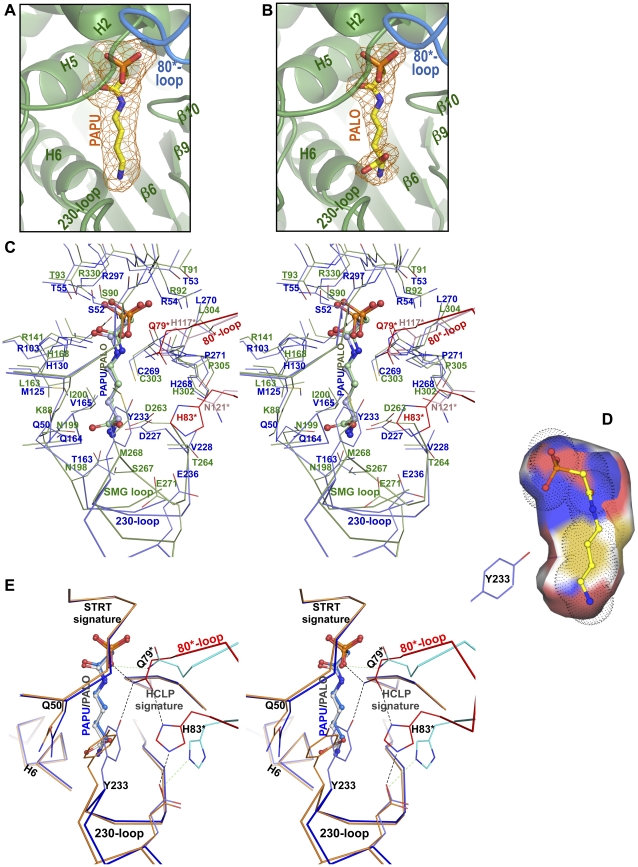
Binding of PAPU and PALO to PTC and comparison with PALO binding to OTC. (A and B) Electron density Fo-Fc omit maps (2.5σ) for PAPU or PALO in the PTC-PAPU (A) or PTC-PALO (B) crystals. The protomer hosting the binding site is shown in green, labeling some nearby secondary structure elements, while the 80-loop* of the neighboring subunit is represented in blue. (C) Stereoview of active centers of PTC (blue) and hOTC (green) with, respectively, PAPU and PALO (represented as balls and sticks). Elements of the 80-loop* of the adjacent subunit is illustrated with C atoms in red and pink for PTC and OTC, respectively. (D) Longitudinal section of the PAPU binding site in PTC, showing in the surface the sulfur, carbon, oxygen and nitrogen atoms of the protein colored in yellow, white, red and blue, respectively. PAPU is shown in ball and stick representation with the Van der Waals spheres of its atoms shown dotted. (B) Stereoview of active centers of PTC-PAPU (blue) and PTC-PALO (orange), with bound PAPU (blue) and PALO (grey) represented as balls and sticks. The 80-loop* of the adjacent subunit is illustrated in red and light blue for PTC-PAPU and PTC-PALO, respectively. Some hydrogen bonds forming a roof over the active center that are affected by the binding of PALO are shown as dashed lines.

The finding in each subunit of the crystals of PTC-PAPU and PTC-PALO of very elongated masses of non-protein electron density that fit extended PAPU and PALO molecules (Figure 7A and 7B), have clarified how can PTC discriminate between ornithine and putrescine. The active centers of PTC and OTC are highly similar (Figure 7C). One significant difference is the replacement by glutamine (Q50) in PTC of a lysine (K88 of hOTC) that is constantly found in OTCs two positions upstream of the CP signature STRT (Figure 7C and S2). This replacement may importantly affect the selectivity for the amine substrate since the lysine found in OTC helps neutralize the carboxylate group of ornithine, which is missing in putrescine (Figure 7C). A second significant change for discrimination between putrescine and ornithine is the replacement in PTC of the SMG loop of OTC by the 230-loop (Figure 7C and S2). The sequence and the conformation of this PTC loop differ importantly from those of the SMG loop, including the replacement of the SMG signature of OTC by the PTC signature ^227^DVWYGLY ^233^ (underlined residues are constant or conservatively replaced in PTCs). The influence of this last loop in the selectivity of PTC for putrescine is illustrated by the interactions with putrescine of Y233. In the narrow and mainly hydrophobic sheath that encircles the putrescine moiety of PAPU (Figure 7D), the phenolic ring of Y233 sits flat as a tile at the site that would be occupied by the α-COO^−^ of ornithine, hampering ornithine but not putrescine binding (Figures 7C–E). This phenolic ring is stabilized in such position by hydrophobic contacts with nearby residues and with the hydrocarbon chain of putrescine, as well as by hydrogen binding of its O atom with the side-chain N atom of Q79* from the 80-loop* of an adjacent subunit (Figure 7E). In contrast, in the PTC-PALO complex the phenolic group of Y233 is far from the ornithine and it interacts extensively with the carbon chain of Q50 (Figure 7E), leaving widely exposed the ornithine moiety of PALO. This loss of extensive contacts with the hydrocarbon chain of the bound ornithine should strongly decrease the stability of the enzyme-ornithine complex and thus the affinity for ornithine, as reflected in the much larger K_m_ value of PTC for ornithine than for putrescine (1.4±0.2 mM for putrescine and 36.4±3.5 mM for ornithine, our own data; see also [7]).

**Figure 8:**
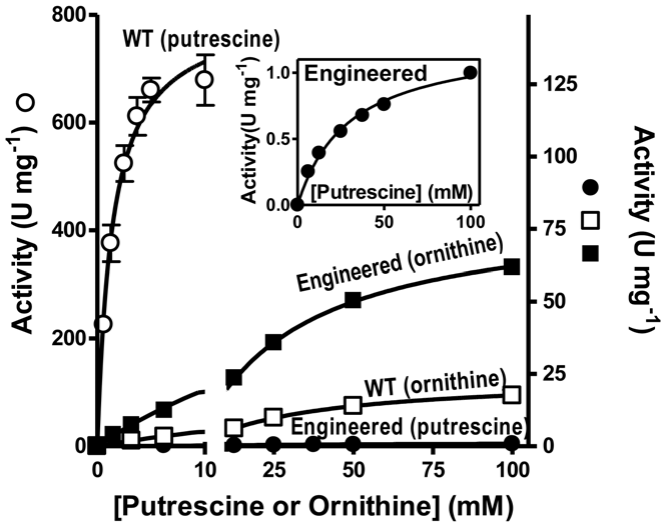
Changes in PTC specificity by engineering the 230-loop. Putrescine (circles) or ornithine (squares) concentration dependency of enzyme activity for wild type PTC (WT, open symbols) and for the engineered form in which the ^230^YGLY^233^ sequence was replaced by VSMG (Engineered, closed symbols). WT activity is plotted in the left y-axis whereas other activities are plotted on the right y-axis. Inset: zoom on the engineered form with putrescine.

A change in the position of Y233 in the PTC-PALO complex may also account for the decreased k_cat_ of PTC for ornithine [7]. In this complex the hydrogen bond between the Y233 phenolic O atom and Q79* is lost and the position of Q79* and of the 80-loop* is changed (Figure 7E). Q79* is crucial for formation by the 80* and 230 loops of a roof over the active center that buries the bound PAPU, and that must be opened and closed in each catalytic cycle to allow substrate access and product release. The roof opening an closing cycle and the catalytic cycle may be synchronized by the hydrogen binding of this N atom of Q79* with an O atom of the phosphonate moiety of PAPU and thus of CP (Fig. 7E). Actually, this hydrogen bond links one subunit with the substrate bound to another subunit, supporting the view that in PTC, as in other transcarbamylases, two subunits are needed for building the active center (Figure 7C). In any case, the roof over bound PAPU involves the cooperation of both subunits. Although the side chain O atom of Q79* is hydrogen-bonded to one N atom of the imidazole ring of H83* belonging to the 80-loop*, the other N atom of this imidazole is bound to the side-chain carboxylate of E236, of the 230-loop of the subunit hosting the active center (Figure 7E). The breaking of the roof may be deleterious for enzyme activity if catalysis requires confinement of the substrates under this closed roof. Indeed, the abolition in the PTC-PALO complex of the hydrogen bond between the phenolic O atom and Q79* also results in the secondary loss of the hydrogen bond linking Q79* and H83* (Figure 7E), tearing open at its center the roof over the active center, clearing the way to allow the escape of ornithine. Actually, of the six PTC subunits in the asymmetric unit of the PTC-PALO complex, the 80-loop is not visible in three of them and the 230-loop in two, indicating mobility or disorder of these loops, reflecting the roof-destabilizing effect of the loss of hydrogen bonds caused by the mispositioning of Y233 resulting from ornithine binding. In contrast, both subunits found in the asymmetric unit of the PTC-PAPU complex have well conformed roof and loops over the bound PAPU. Since the residence time of the substrates in the active center might determine the probability of reaction, the tearing or opening of the roof in the complex with ornithine may decrease this residence time and reduce the V_max_ with ornithine, as observed experimentally (813±38 U/mg for putrescine and 24±1 U/mg for ornithine, Figure 8).

### Engineering of the 230-loop of PTC favors the use of ornithine and impairs that of putrescine

We tested the effect of replacing the sequence ^230^YGLY^233^ of the putrescine signature of *E. faecalis* by its OTC counterpart VSMG. The engineered enzyme, produced recombinantly and purified similarly to the wild-type enzyme, exhibited a dramatic reduction in its activity with putrescine (Figure 8). This reduction was due to a drastic reduction in the velocity at saturation of putrescine (from 813±38 U/mg to 1.13±0.13 U/mg) together with a ∼20-fold increase in the apparent K_m_ for putrescine (from 1.41±0.22 mM to 32.9±8.8 mM) (Figure 8). These findings highlight the importance of this loop for putrescine binding and for determining the rate of the reaction. In contrast with this impairment of the ability of the enzyme to use putrescine, the engineered enzyme was faster with ornithine than wild-type PTC, mainly because of a ∼3.5-fold increase in the velocity at saturation of ornithine (from 24±1 U/mg for wild-type PTC to 82±3 U/mg for the engineered enzyme), with only a minor decrease in the apparent K_m_ value for ornithine (from 36.4±3.5 mM to 32.0±2.0 mM) (Figure 8). In summary, this change in the loop decreased the catalytic efficiency (V_max_/K_m_) of the enzyme for putrescine from a value of 577 U mg^−1^ mM^−1^ to 0.03 U mg^−1^ mM^−1^, whereas it increased the catalytic efficiency for ornithine from a value of 0.67 U mg^−1^ mM^−1^ to 2.6 U mg^−1^ mM^−1^, rendering the enzyme a better ornithine transcarbamylase than putrescine transcarbamylase. These results confirm the importance of the 230-loop in the discrimination between putrescine and ornithine.

### A structure-based simple signature for PTC identification from its sequence

For many years the gene for PTC could not be differentiated in genomic databases from that of OTC. However, bibliographic datamining led to the rediscovery [20] of the N-terminal sequence of *E. faecalis* PTC [31], allowing identification of the PTC gene in this organism, and by sequence alignment, in other organisms [20], [32]. From the comparison of these sequences, 5 long motifs were proposed as characteristic of PTCs [20]. By using the present structural information we can provide a simplified signature for PTC identification. PTC sequences can be recognized because they have the invariant ^52^STRT and ^268^HCLP (numbering is for *E. faecalis* PTC) sequences for, respectively, CP and ornithine/putrescine binding (shared also by OTCs) but they lack the lysine found in OTCs two residues upstream of the ^52^STRT sequence, a lysine that is involved in OTCs in ion pairing with the α-COO^−^ group of ornithine. In addition, about 40 residues upstream of the invariant HCLP sequence of PTCs and OTCs, the characteristic XSMG sequence of OTCs that gives its name to the SMG loop of these enzymes is replaced in PTCs by the specificity-determining 230 loop sequence ^230^(Y/W)(G/W)(V/L/I)X. Additional characteristic traits of PTCs are provided by the finding of a ∼20-residue C-terminal extension when the sequence is aligned with that of an OTC, and by the finding within this extension of the signature identified above in the C-terminal helix, ^330^(L/f/v/i)XX(F/Y/L/M/v)(L/i/m).

### Final remarks

The incomplete differentiation between putrescine and ornithine by PTC, and the close similarity of the sequences as well as the structures of PTC and OTC [20] indicate that these two enzymes are highly related, possibly having diverged relatively recently [9]. The time from divergence may have been too short to allow PTC to perfect a strict specificity for putrescine. This recent divergence of PTC and OTC contrast with the much more extensive and, most likely, much earlier divergence of two other components of the same operon, the agmatine deiminase (AgDI) and agmatine/putrescine antiporter, from the corresponding components of the analogous arginine deiminase (ADI) operon of arginine catabolism (Figure 1A and B) [9]. Thus, it appears unlikely that the AgDI pathway arose from an “en bloc” duplication of the operon for the ADI pathway, favoring the view that the AgDI gene cluster was pasted together from its isolated components. The essentially identical gene composition and organization of this gene cluster in the organisms in which a PTC has been annotated [19], [20], [31], [32] indicates a relatively recent single-time origin of the cluster, which has.spread to relatively few organisms compared with the more widespread ADI gene cluster (see for example the Comprehensive Microbial Resource of JCVI, http://cmr.jcvi.org/tigr-scripts/CMR). This restricted distribution perhaps reflects a recent origin (less time to spread) and the more limited occurrence of agmatine than arginine in bacterial environments (see for example [33]).

Given the particularly close similarity in sequence and structure of pfOTC and PTC, it is tempting to propose that both share a hyperthermophilic archaeal ancestry. The relatively low specific activity of PTC (∼700 U/mg at 37°C) compared with typical OTCs (∼4000 U/mg at 37°C [34], [35]) may be a reminiscence of a hyperthermophilic origin, since typically hyperthermophilic enzymes have low specific activities at mesophilic temperatures [36], as is the case for pfOTC [37]. A hyperthermophilic origin could fit the need for the extra (relative to all other transcarbamylases) C-terminal helix (helix 13) found in PTC. The pfOTC trimer, when isolated from its dodecamer which is essential to give this enzyme its resistance to high temperatures [21], [38], is predicted to have relatively low stability (ΔG for dissociation, 12.8 kcal/mol, estimated with the PISA server), compared with typical mesophilic OTC trimers (for example, estimated ΔG for dissociation of hOTC, 26.4 kcal/mol). In the pfOTC dodecamer the trimer is stabilized by the links between subunits from adjacent trimers through hydrophobic contacts and ion-pairs mainly mediated by helix 1 [21]. Therefore, to make posible its existence as an isolated trimer, the potential hyperthermophilic ancestor of PTC had to replace helix 1-mediated intertrimeric contacts by contacts mediated by the novel subunit-subunit interlinking C-terminal helix (Figure 5A). In this way, helix 13 plays the dual role of stabilizing the trimer and of preventing supratrimeric association.

If PTC derives from an ancestral hyperthermophilic enzyme having a dodecameric architecture, as pfOTC, it is uncertain which was the selective pressure for eliminating this supratrimeric organization, particularly since a change to trimeric architecture decreases the stability of the trimer, making necessary to incorporate the C-terminal helix for increasing stability. Perhaps such type of organization could restrict domain movements, known to be important in transcarbamylases including OTC [3], [28], [39]. However, in pfOTC the dodecameric architecture does not appear to hinder the approach of the C-terminal domain to the N-domain that is associated with catalysis [40]. Another disadvantage of a stable supratrimeric organization could be a tendency to *en bloc* degradation of the whole oligomer when one of the trimers forming it is damaged as per oxidative or proteolytic damage. Perhaps the most attractive reason against supratetrameric organizations would be if this organization would not be compatible with formation of a metabolon involving AgDI, PTC and the agmatine/putrescine antiporter at the bacterial membrane. Such complex would make sense for highly efficient fermentation of the agmatine in the medium, which would be used as soon as it is internalized. Agmatine is a precursor of polyamines [41], and the restriction of its fermentative use to the agmatine crossing the bacterial membrane would prevent affectation of intracellular polyamine pools and even the fermentative consumption of endogenously generated agmatine. The participation of PTC in a supramolecular complex would not be an exceptional case among transcarbamylases. In some bacteria aspartate transcarbamylase makes a complex with the next enzyme in the pyrimidine biosynthesis pathway [42], and in animals it belongs to a large multifunctional protein (CAD) catalyzing the initial three steps of pyrimidine synthesis [43].

Whichever the reasons for choosing a trimeric rather than a supratrimeric organization in PTCs, the use of novel secondary structure elements such as the C-terminal helix of PTC to prevent oligomerization is not without precedent, having been reported, for example, in type III aspartokinase [44]. This enzyme incorporated a two-helix hairpin that covers a surface that is used in all other known members of the amino acid kinase family for interaction with another subunit. In this way, this novel helix hairpin prevents intersubunit contacts via this surface [44]. Although the C-terminal helix of PTC appears restricted exclusively to this type of transcarbamylase, the structure of hOTC suggests that a helix 1-shielding strategy has been used by this enzyme for avoiding supratrimeric oligomerization. hOTC has a shorter (relative to PTC) C-terminal extension [24] that is not projected towards the adjacent subunit but that crosses transversally helix 1 of the same subunit, thus being expected to prevent intertrimeric interactions (Figure 5B, 2nd panel from right and detail below it). This finding, and that in PTC of a C-terminal helix that prevents supratrimeric oligomerization renders important to examine further the roles of supratrimeric organizations among transcarbamylases.

## Materials and Methods

### Wild type and engineered enzymes

Pure recombinant *E. faecalis* PTC having the C-terminal His_6_ extension SAAKLAAALEH_6_ was prepared, and *E. faecalis* OTC (specific activity, 4021 U/mg) was obtained, as reported [9]. PTC carrying the R54G mutation was prepared from pET-PTC [9], using the Quickchange site-directed mutagenesis kit (from Stratagene) and the oligonucleotide pair 5′-AATCTTCAACTGGCACACGAGTATCTTTTGAAAC-3′ and 5′-GATACTCGTGTGCCAGTTGAAGATTGTTGGAAA-3′ (underlining indicates mutated bases). The same strategy was used for changing the sequence of the 230-loop form ^230^YGLY to ^230^VSMG, using as mutagenic primers 5′-CGGATGTTTGGGTGTCCATGGGTGAAGCGGAATTATCTGAAGAA-3′ and 5′-AATTCCGCTTCACCCATGGACACCCAAACATCCGTGTACAAC-3′. The presence of these mutations was confirmed by sequencing. The procedure for expression and purification of these mutant proteins, and their final purities (assessed by SDS-PAGE) and yields were similar to those for wild-type PTC.

For deletion of the C-teminal helix of PTC, the region of the *ef0732* gene encoding amino acids 1–317, followed by a stop codon, was PCR-amplified from *E. faecalis* SD10 genomic DNA [9] using a high-fidelity thermostable DNA polymerase (Deep Vent; New England Biolabs) and the primer pair 5′ TACTTCCAATCC**ATGAAAAGAGATTACGTTAC-3′** and 5′ TATCCACCTTTACTGTCA **AATGCTTTGAGTGTCTAAG-3′** , (bold-type, PTC-coding sequences, underlined, stop codon). The product was incorporated in the cloning vector pNIC28-Bsa4 (a gift from O. Gileadi, Oxford University, UK) by ligation-independent cloning using T4 DNA polymerase [45]. The resultant plasmid carrying the truncated gene, isolated from DH5α cells and confirmed by sequencing to carry the correct construct (including a sequence that encodes the N-terminal His_6_ tag MH_6_SSGVDLGTENLYFQS) was transformed into BL21 (DE3) cells (from Novagene) that were co-transformed with pGroESL. The latter plasmid is a pACYC184-derived expression plasmid (provided by A. E. Gatenby, DuPont de Nemours, Wilmington, DE) that encodes the *E. coli* chaperonins GroES and GroEL [46]. After growth of the cells at 37°C to ∼0.7 OD_600_ in liquid LB supplemented with kanamycin (0.04 mg/ml) and chloramphenicol (0.035 mg/ml), 0.1 mM isopropyl ß-D-thiogalactopyranoside was added and the culture was continued 36 hours at 20°C. Cell harvesting, disruption and purification of the protein at 4°C using Ni-based chromatography were as for the wild-type enzyme [9].

### Crystallization and data collection

The crystals of PTC-PAPU, of ∼0.3 mm largest dimension and hexagonal prismatic habit, were obtained as reported [9] at 294 K by the vapor diffusion technique in hanging drops prepared in 24-well plates by mixing 1 µl of a crystallization solution composed of 125 mM (NH_4_)_2_SO_4_, 17% polyethylene glycol (PEG) 3.35 K (from Hampton Research), 0.1 M bis-Tris, pH 5.5, and 1 µl of 10-mg ml^−1^ PTC in 50 mM Tris-HCl, pH 7.5 containing 0.43 mM PAPU [9]. Crystals of wild-type and truncated PTC with PALO were obtained at 21°C by vapor diffusion in 96-well plates, in 0.8 µl sitting drops dispensed with a HoneyBee X8 robot (Genomics Solutions) using commercial screens (from Hampton Research and Jena Bioscience). The best crystals for the wild type enzyme (maximal dimension, 0.1 mm, and similar habit to the PTC-PAPU crystals) were obtained in 1/1 drops of a solution of PTC (7.5 mg ml^−1^) in 50 mM Tris-HCl pH 7.5 containing 1 mM PALO (a gift of D. Shi, Children's Research Institute, Washington DC) and of a crystallization solution containing 0.2 M NH_4_ acetate, 25% PEG 3.35 K, 0.1 M bis-Tris, pH 5.5. The best crystals for truncated PTC (maximal dimension 0.2 mm) were obtained using a crystallization solution consisting of 0.2 M MgCl_2_, 30% PEG 0.4 K, 0.1 M Hepes, pH 7.5. While for freezing PTC-PAPU and PTC-PALO crystals the cryobuffer was reservoir solution enriched with 15% or 20% glycerol, respectively, the truncated PTC-PALO crystals were flash-cooled in liquid nitrogen without cryoprotectant.

The crystals were diffracted at 100 K (Oxford Cryo-Systems) (Table 1) using synchrotron radiation (ESRF, Grenoble, France; beamlines are detailed in Table 1). The dataset for PTC-PAPU was processed and scaled with DENZO and SCALEPACK (HKL-2000 program package [47]) and imported to CCP4 [48] with SCALEPACK2MTZ and TRUNCATE (CCP4 suite [48]). The data for the other two crystals were processed with MOSFLM, SCALA and TRUNCATE (CCP4 suite [48]). Data collection and refinement statistics are shown in Table 1.

### Phasing, model building, and refinement

Molecular replacement using MOLREP (CCP4 suite [48]), utilizing a polyalanine model of the subunit of *Pyrococcus furiosus* OTCase (PDB file 1A1S, [21]) yielded a satisfactory solution for the PTC-PAPU crystal, consisting of two subunits in the asymmetric unit. Rigid-body and restrained refinements were performed using REFMAC5 [49], alternating with graphic model-building sessions with the program COOT [50]. B-factors and positional non-crystallographic symmetry restraints were used and gradually released as refinement progressed. All the diffraction data were used throughout the refinement process except the 5% randomly selected data used to calculate the *R*_free_ [51]. Refinement converged to a final *R*-value of 18.7% (*R*_free_ = 21.6%). The final model, at 2.5-Å-resolution, consisted of two chains, each one encompassing residues 1 to 340 and containing a bound molecule of PAPU. The stereochemistry of the model, checked with PROCHECK [52], was good, with only two residues (Met125 and Leu270) in the disallowed region. Table 1 summarizes the data on the refinement process and on the final model. PAPU was built in the non-protein density observed at the active center, by taking one PALO molecule from the structure of *E. coli* OTC bound to PALO (PDB accession number 2OTC), then removing the carboxylate group of the ornithine moiety to build the PAPU, and then placing this molecule manually within the observed electron density, finally refining ligand and protein with REFMAC5. TLS was used in the last steps of refinements [53] using the TLSMD server (http://skuld.bmsc.washington.edu/~tlsmd/, [54]) for choosing the TLS groups.

For the PTC-PALO and the truncated PTC-PALO crystals, molecular replacement was performed with MOLREP using the PTC-PAPU devoid of PAPU as a model. Helix 13 was removed from the model for phasing of the truncated protein crystal. The number of subunits in each asymmetric unit is given in Table 1. Model building and refinement were carried out as for PTC-PAPU. One molecule of PALO per subunit was found in the PTC-PALO complexes. *R*, *R*_free_ values and the stereochemistry were excellent in the two cases, with the same two residues as above in the disallowed region.

### Other methods

PTC activity was assayed as previously described [9] at 37°C in 50 mM Tris-HCl pH 7 in the presence of 10 mM putrescine and 10 mM CP, determining carbamylputrescine production. The OTC activity of PTC was determined in the same way, replacing putrescine by ornithine and using the Tris-HCl at pH of 8.5. With both activities, when the concentration of one substrate was varied, the other was kept constant at 10 mM. Substrate concentration-activity data were fitted to hyperbolae using program GraphPad Prism (GraphPad Software, San Diego, Calif). One enzyme unit corresponds to the production of 1 µmol carbamylputrescine min^−1^.

Analytical size exclusion chromatography of PTC (wild-type o truncated) was performed by injecting 20 µl of a 10 mg/ml solution of the protein to a Superdex 200HR 5/150 GL column fitted in an Åkta FPLC and equilibrated and eluted (flow rate, 0.25 ml min^−1^) at 24°C with 50 mM Hepes pH 7.5, 0.25 M NaCl, monitoring optical absorption at 280 nm. The column was calibrated with the following protein standards of known mass (given in kDa): thyroglobulin (669), ferritin (440), amylase (223.8), *Thermotoga maritima* acetylglutamate kinase (182), bovine serum albumin (66.4), carbonic anhydrase (29) and ribonuclease (13.7).

Protein was assayed by the method of Bradford [55], using a commercial reagent from Bio-Rad and bovine serum albumin as a standard. SDS-PAGE was carried out as described by Laemmli [56]. Sequence alignments were carried out with ClustalW [57], using default values. Superposition of structures was carried out with the program SSM [26]. Buried surface areas were calculated using NACCESS (http://wolf.bms.umist.ac.uk/naccess). Figures of protein structures were generated using Pymol (http://pymol.sourceforge.net/).

### Atomic coordinate and structure factor accession number

The coordinates and structure factors have been deposited in the Protein Data Bank (http://www.rcsb.org/) with the accession codes 4A8H, 4A8P and 4A8T for PTC-PAPU, PTC-PALO and truncated PTC-PALO, respectively.

## Supporting Information

Figure S1 **Superimposition o PTC and hOTC trimers.** Stereoview of Cα trace of *E. faecalis* PTC (blue) and human OTC (green) trimers viewed along the 3-fold axis. PAPU and PALO are depicted in ball and stick representation.
(TIF)

Figure S2 **Alignment of PTC and hOTC sequences and structures.** Horizontal arrows and open cylinders above and below the sequences denote respectively α-helices and β-strands. Since each subunit interacts with two different subunits in the trimer, triangles and circles indicate residues interaction with one and the other neighboring protomers, respectively, whereas squares denote residues that contact both adjacent protomers. These symbols are open when these contacts only take place in PTC, and filled when they interact both in PTC and OTC. Grey-filled rhombi denote residues that interact with PAPU in PTC-PAPU and with PALO in hOTC, whereas open rhombi only interact with the bisubstrate analog in PTC. Vertical arrows indicate residues corresponding to those involved in intertrimeric interactions in pfOTC (black-bordered) or in *L. hilgardii* (blue-filled). Asterisks denote identity between the sequences shown. White residues on black backgrounds are those invariant among PTCs or among OTCs, whereas those on grey background exhibit conservative replacement within one or the other transcarbamylase family. The STRT, HPXQ and HCLP motifs are encased in squares colored pink, blue and orange, respectively. The mitochondrial signal peptide is underlined in the hOTC sequence. The OTC sequences that were aligned correspond to the following Uniprot accession numbers: P00480, P08308, P44770, P21302, P04391, Q8XCB8, Q08016, Q8EVF5, P59779, P96108, P68746, P75473, Q59283, P0A5M9, Q55497, P11724, Q02047, Q43814, Q48296, P96172, P18186, Q51742, P96134, Q5829, P11066, Q00291, P11803, P14995, P05150, P31317, P31326, P11725, P00481 and P00480). The PTC sequences that were aligned correspond to the following Uniprot accession numbers Q837U7, C8WMM1, Q8DW19, D2P024, Q03HM9, C0XJB3, E8M734, Q6MSR6, B7S4N1, C9QF99, D0AI19, Q03NG2, C0WN35, Q38ZL1, Q2SRJ4, F8K6E0, Q03HM9; and to the following NCBI entries: YP_003143629.1, YP_133569.1, YP_001684585.1, YP_001412420.1, ZP_08712612.1, ZP_08723343.1, YP_001412420.1 and YP_003181041.1.
(TIF)
