# Supplementary figures and images for: New Insight into the Transcarbamylase Family: The Structure of Putrescine Transcarbamylase, a Key Catalyst for Fermentative Utilization of Agmatine
Source: PLoS One. 2012 Feb 20;7(2):e31528. doi: 10.1371/journal.pone.0031528 (PMC3282769; doi:10.1371/journal.pone.0031528)

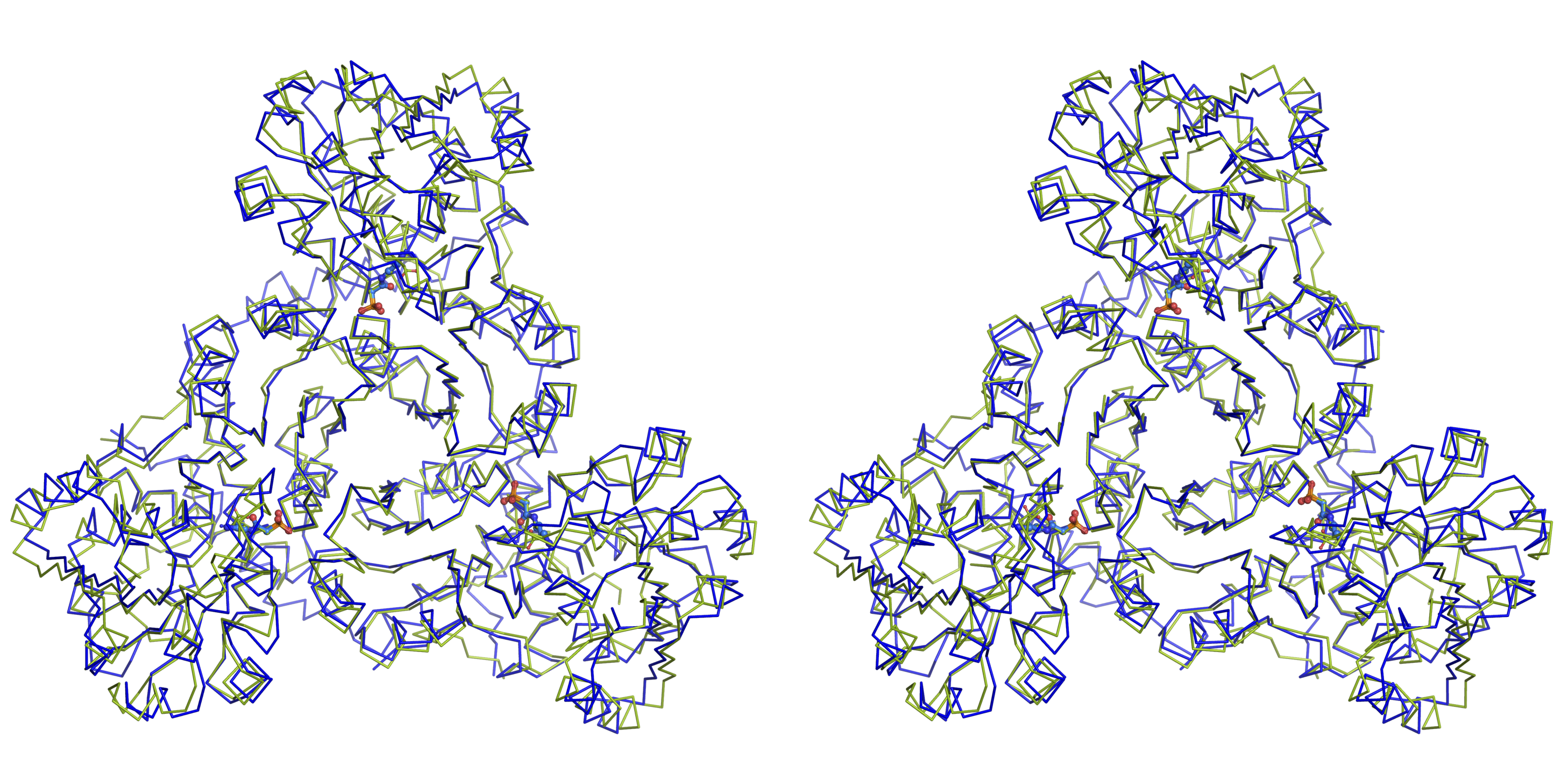

Supplement: Figure S1 — Superimposition o PTC and hOTC trimers. Stereoview of Cα trace of E. faecalis PTC (blue) and human OTC (green) trimers viewed along the 3-fold axis. PAPU and PALO are depicted in ball and stick representation. (TIF) [file pone.0031528.s001.tif]

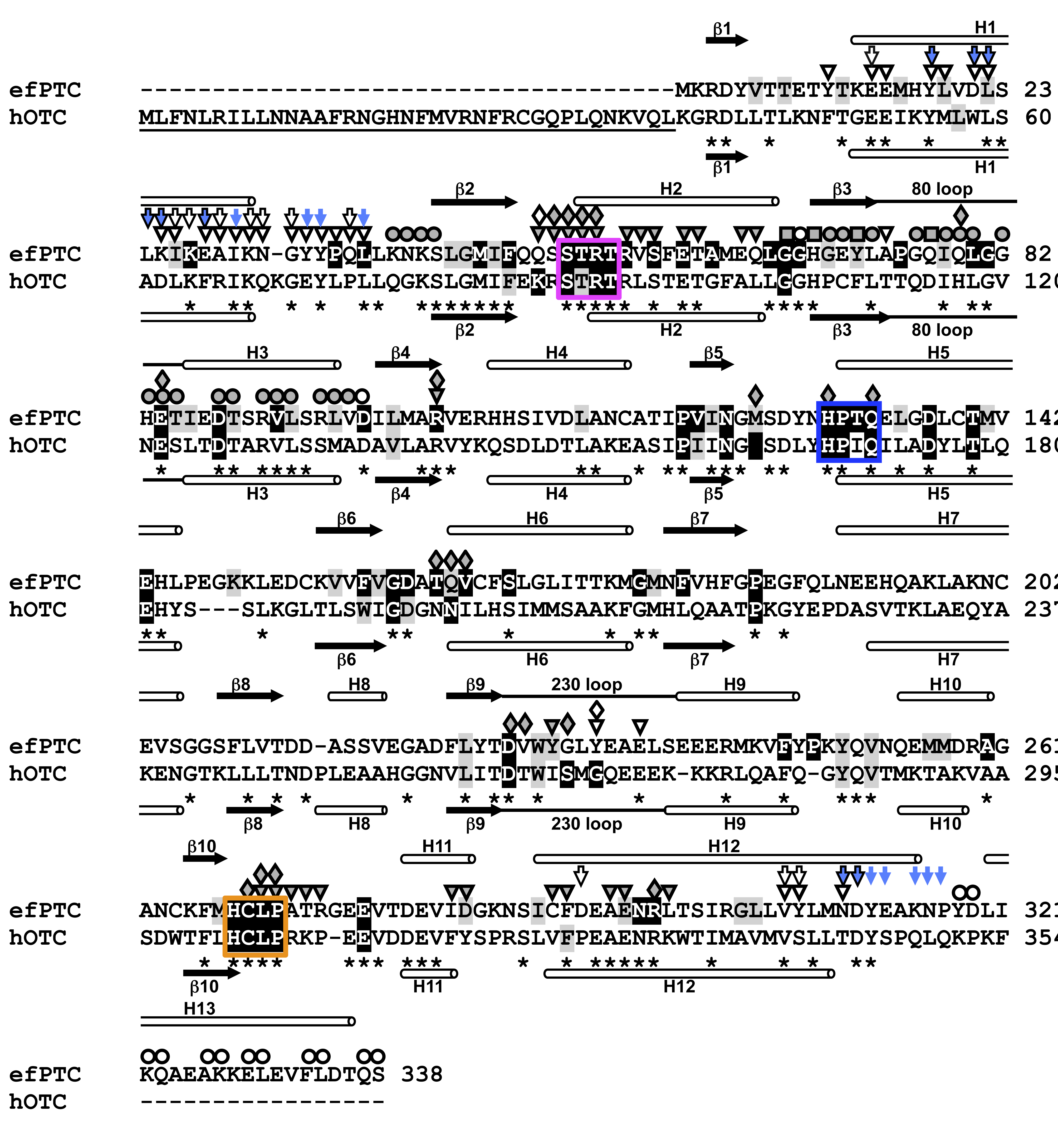

Supplement: Figure S2 — Alignment of PTC and hOTC sequences and structures. Horizontal arrows and open cylinders above and below the sequences denote respectively α-helices and β-strands. Since each subunit interacts with two different subunits in the trimer, triangles and circles indicate residues interaction with one and the other neighboring protomers, respectively, whereas squares denote residues that contact both adjacent protomers. These symbols are open when these contacts only take place in PTC, and filled when they interact both in PTC and OTC. Grey-filled rhombi denote residues that interact with PAPU in PTC-PAPU and with PALO in hOTC, whereas open rhombi only interact with the bisubstrate analog in PTC. Vertical arrows indicate residues corresponding to those involved in intertrimeric interactions in pfOTC (black-bordered) or in L. hilgardii (blue-filled). Asterisks denote identity between the sequences shown. White residues on black backgrounds are those invariant among PTCs or among OTCs, whereas those on grey background exhibit conservative replacement within one or the other transcarbamylase family. The STRT, HPXQ and HCLP motifs are encased in squares colored pink, blue and orange, respectively. The mitochondrial signal peptide is underlined in the hOTC sequence. The OTC sequences that were aligned correspond to the following Uniprot accession numbers: P00480, P08308, P44770, P21302, P04391, Q8XCB8, Q08016, Q8EVF5, P59779, P96108, P68746, P75473, Q59283, P0A5M9, Q55497, P11724, Q02047, Q43814, Q48296, P96172, P18186, Q51742, P96134, Q5829, P11066, Q00291, P11803, P14995, P05150, P31317, P31326, P11725, P00481 and P00480). The PTC sequences that were aligned correspond to the following Uniprot accession numbers Q837U7, C8WMM1, Q8DW19, D2P024, Q03HM9, C0XJB3, E8M734, Q6MSR6, B7S4N1, C9QF99, D0AI19, Q03NG2, C0WN35, Q38ZL1, Q2SRJ4, F8K6E0, Q03HM9; and to the following NCBI entries: YP_003143629.1, YP_133569.1, YP_001684585.1, YP_001412420.1, ZP_08712612.1, ZP_08723343.1, YP_001 [file pone.0031528.s002.tif]
